# Do Personality Traits Moderate the Effects of Cohabitation, Separation, and Widowhood on Life Satisfaction? A Longitudinal Test for Germany

**DOI:** 10.1007/s10902-022-00573-8

**Published:** 2022-11-02

**Authors:** Wilfred Uunk, Paula Hoffmann

**Affiliations:** 1 Department of Sociology, University of Innsbruck, Innsbruck, Austria; 2 Department of Sociology, Utrecht University, Utrecht, the Netherlands

**Keywords:** Life satisfaction, cohabitation, separation, widowhood, personality

## Abstract

The start and end of a romantic relationship are associated with substantial changes in life satisfaction. Yet, whether Big Five personality traits moderate these relationship transition effects is hardly known. Such knowledge helps to understand individual variation in relationship transition effects and provides the possibility to further test the stress and social support explanations of these effects. Our fixed effects regressions on 28 waves of the German Socio-Economic Panel 1991-2018 show that Big Five traits moderate the effects of relationship transitions on life satisfaction to a limited extent. More neurotic men display a more negative effect of separation, and more neurotic and more agreeable women reveal a more negative effect of widowhood on life satisfaction. Big Five traits do not moderate the effect of the start of cohabitation on life satisfaction. Our findings support the stress perspective of relationship transition effects most and identify emotionally unstable individuals as a particularly vulnerable group.

**Supplementary information:**

The online version contains supplementary material available at 10.1007/s10902-022-00573-8.

## Introduction

Studies have shown that the start and end of a romantic relationship are associated with substantial changes in life satisfaction and other measures of well-being. While life satisfaction generally increases with cohabitation and marriage (Lucas et al., 2003), life satisfaction decreases with separation, divorce, and widowhood (Amato, 2010; Hewitt et al., 2012; Lucas et al., 2003). These changes in life satisfaction due to (un-)married cohabitation, separation, or widowhood—we speak of relationship transition effects here—are temporal yet nevertheless pronounced (Baxter & Hewitt, 2011; Lucas et al., 2003; Luhmann et al., 2012; Yap et al., 2012).

Two theoretical perspectives are generally used to explain relationship transition effects on well-being and life satisfaction: the social support perspective and the stress perspective (cf. Amato, 2010; Lucas et al., 2003). The social support perspective explains relationship transition effects by changes in social contacts and support, specifically by an increase in social contacts and support with the start of cohabitation and a decrease with the end of cohabitation. The stress perspective focuses on the end of cohabitation and explains its negative effect on life satisfaction by increased stress. The two perspectives are also used to explain variation in relationship transition effects on life satisfaction across groups. Due to lower levels of social support, men, for example, show stronger increases in life satisfaction from cohabitation and marriage than women and greater losses after separation and divorce (Amato, 2010; Leopold, 2016; Liebler & Sandefur, 2002; Stronge et al., 2019). Furthermore, individuals who experience stress and conflict during the marriage suffer less in life satisfaction after divorce—often even reporting improved happiness—than individuals who do not experience such conflict (Amato & Hohmann-Marriot,^2007^ ).

This paper aims to expand the literature by investigating personality as another potential moderator of relationship transition effects on life satisfaction, an idea referred to as the “person-environment interaction” (Yap et al., 2012: 478). Knowing whether and how personality traits moderate relationship transition effects is important for several reasons. Firstly, it contributes to the theoretical understanding of the mechanisms underlying relationship transitions and provides an indirect test of the social support and stress explanation. The two perspectives are generally hard to disentangle because both expect negative consequences of the end of a relationship on life satisfaction. Yet, regarding the moderation by personality traits, expectations differ. While the social support perspective expects more negative effects of separation and widowhood for more introverted and neurotic individuals (due to a lower active social life and lower social support), the stress perspective only expects more negative effects for more neurotic individuals (due to more stress). Secondly, investigating the moderating effects of personality traits may help understand individual differences in reactions to relationship transitions (Yap et al., 2012). The socio-demographic, psychological, and well-being literature has chiefly focused on the main effects of personality and relationship transitions on well-being and life satisfaction but neglected possible interactive effects. Thirdly, and lastly, from a societal perspective, it is important to identify which individuals suffer more from separation and widowhood than others. More awareness and specific intervention programs might support vulnerable groups to better cope with the adverse situation.

The moderating influence of personality traits on the relationship-life satisfaction link is empirically hardly tested, and findings are partially inconsistent. Boyce et al., (2016) found for Germany that conscientious and introverted women experience greater life satisfaction following marriage than their counterparts, but observed that among men it is the extraverted who experience greater benefits following marriage. Pai & Carr (2010) found for the U.K. that the effect of late-life spousal loss on depressive symptoms was smaller for highly extraverted and conscientious individuals, but noted that the protective effects of personality traits depend on the expectedness of the death. Yap et al., (2012) for the U.K. and Anusic et al., (2014) for Australia, on the other hand, did not find that personality traits moderated the effects of marriage and widowhood on life satisfaction and affect.

We enrich the above studies on the moderating influence of personality traits on relationship transition effects by (a) using a more extensive set of person-year panel data, enhancing the number of relationship transitions analyzed; (b) applying fixed effects regression techniques, improving the estimation of relationship transitions effects on life satisfaction (cf. Amato, 2010); and (c) focusing on changes in cohabitation status rather than marital status (i.e., the start of cohabitation and the end of cohabitation through separation or widowhood). The latter is an improvement because the presence of a partner in the household is more consequential for life satisfaction than legal marital status (cf. Brown, 2004). We specifically focus our analyses of Big Five traits on neuroticism and extraversion. A recent and extensive meta-analysis by Anglim and colleagues (2020) shows that neuroticism and extraversion are stronger associated with life satisfaction than the other three Big Five traits (conscientiousness, agreeableness, and openness). In addition, the social support and stress perspectives differ in their expectations regarding the moderating influence of neuroticism and extraversion on relationship transition effects.

## Theory and Hypotheses

We use the abovementioned social support and stress perspective to explain relationship transition effects on life satisfaction and enhance these perspectives with assumptions from the Big Five theory to derive hypotheses on the moderating influence of personality traits.

### Social Support Perspective

The social support perspective expects that the start of a romantic relationship increases life satisfaction and that the end of a romantic relationship decreases life satisfaction (DeMaris, 2018; Thomas, 2010; Siedlecki et al., 2014; Stronge et al., 2019). One reason for this expectation is that a partner provides social support (Amato, 2010). Social support refers to an individual’s experience of feeling loved, valued and cared for by others. Informational support (sharing information or advice), instrumental support (material aid), and emotional support are often defined as sub-types (Taylor, 2011).

Another reason why a romantic relationship may increase life satisfaction is that a new social network is available through one’s partner. These new social contacts enhance life satisfaction, and their loss—through relationship end—may decrease life satisfaction. The social support perspective further expects that the end of a relationship through widowhood has more negative effects on life satisfaction than separation (Soulsby & Bennett, 2015). This is because separation often occurs due to a lack of commitment within (married) cohabitation. In addition, the social support perspective helps to explain variation in relationship transition effects by subgroup. Men, for example, profit disproportionally from having a romantic relationship (Amato, 2010) because they have fewer (emotionally supporting) social contacts than women (Liebler & Sandefur, 2002), and the partner is more often their primary source of social and emotional support (Taylor, 2011). Conversely, men also suffer more from the end of a partnership than women due to the loss of companionship and mutual and ex-partner’s social contacts (Terhell et al., 2004).

It is important to note that within the social support perspective, two views exist on how social support affects well-being (Taylor, 2011). The first view, the direct effect hypothesis, assumes that social support immediately benefits (mental) well-being. The second view, the buffering hypothesis, views social support as a resource that reduces burden in times of distress. Having social support, an individual can better cope with an adverse situation and thus maintain higher well-being levels. Both hypotheses have been supported in the literature (Ibid).

### Stress Perspective

The stress perspective focuses on the end of cohabitation and its adverse effects on life satisfaction. The explanation does not lie in the loss of social support and social contacts but increased stress (although these two factors are interlinked; Hewitt et al., 2012; cf. Amato, 2010). Separation and widowhood are associated with large-scale changes in the life of individuals, such as the bereavement of an important confidante and the loss of resources. According to the stress theory (Pearlin et al., 2005), such changes enhance distress levels and reduce well-being. Moreover, the conservation of resource theory postulates that the threat of resource loss (e.g., of a shared home and common possessions) increases stress in addition to the actual loss of resources (Hobfoll, 1989).

The stress perspective also expects variation in separation effects across groups. For example, the negative effect of separation on life satisfaction may be stronger for those who experience high conflict in and after the separation process (Smock et al., 1999). In addition, the adverse effect of separation may be weaker, even showing improved happiness, for those who experience stress and conflict during the marriage (Amato & Hohmann-Marriot, 2007).

### Big Five Theory and Expectations on the Moderation by Personality Traits

The Big Five theory is employed as an additional theoretical framework to derive predictions on the moderating effects of personality on the relationship-life satisfaction link. The Big Five theory, also called the Five-Factor Model, is well established in the scientific community and achieved high consensus among scholars (Larsen & Buss, 2018). The fundamental model assumption is that individual differences in personality cause differences in personal perception and behavior. Therefore, personality influences a person’s base level of well-being (Cummins, 2016) as well as the reaction to important life events (Bonanno, 2004; Eysenck & Eysenck, 1985).

The model identifies five basic personality dimensions: openness, conscientiousness, extraversion, agreeableness, and neuroticism (Costa & McCrae, 1985). Among these five dimensions, extraversion and neuroticism are central to subjective well-being, especially to the cognitive dimension of well-being (i.e., life satisfaction; Anglim & Grant 2016; Busseri & Sadava, 2011; Schimmack et al., 2002; Schneewind & Gerhard, 2002). While neuroticism is associated with lower life satisfaction (Steel et al., 2008; Costa & McCrae, 1980), extraversion is associated with greater happiness, overall affect, and quality of life (Lucas et al., 2008).

The traits neuroticism and extraversion can be described as follows: neurotic people tend to worry a lot and show higher levels of guilt- and anxiety-proneness, are moody, unstable, pessimistic, and complaining. They tend to overreact to frustration or difficult situations, get irritated quickly and need a longer time to calm down to the initial state after being upset (Costa & McCrae, 1980; Larsen & Buss, 2018). Extraverts enjoy frequent social interaction and talking to people. They like going to parties, seeking social attention, having high levels of commitment, and engaging in risky behavior. The words that describe individuals scoring high on the extraversion scale are sociable, forward, assertive, and adventurous. People found on the other end of the extraversion scale, the introverted like to spend more time on their own, often have a small circle of close friends, and can be described as distant and aloof. At the same time, introverted people prefer a routinized and predictable way of living (Larsen & Buss, 2018).

Because neuroticism and extraversion influence individual perceptions, these personality traits may also lead to distinct responses to life events. People scoring higher on the extraversion scale are generally more positive about enjoyable events and less negative about non-enjoyable events than people scoring lower on the extraversion scale. The contrary is true for those scoring high on the neuroticism scale. They are less positive about enjoyable events and more negative about non-enjoyable events (Bonanno, 2004; Eysenck & Eysenck, 1985; McCrae & Costa, 1986; Vollrath et al., 1999). If so, one may expect from the Big Five theory that the positive effect of the start of cohabitation on life satisfaction will be weaker (less positive) for more neurotic and introverted individuals than for others (H1). In addition, one may expect that the negative effect of separation and widowhood on life satisfaction will be stronger (more negative) for more neurotic and introverted individuals than for others (H2). For neuroticism, this can also be expected from the stress perspective. Due to their worrying character and greater tendency to overreact to difficult situations, neurotic individuals will experience more stress from separation and widowhood than emotionally more stable persons and more difficulty handling this stress. Since we do not assume augmented stress levels for introverted individuals, the stress perspective only forwards that the negative effect of separation and widowhood on life satisfaction will be stronger (more negative) for more neurotic individuals than for others (H3).

From the social support perspective, one can expect partly overlapping and partly competing hypotheses on the moderating influence of personality traits. To start with, the model provides an alternative explanation of the association of personality traits with life satisfaction. More neurotic and introverted people report lower rates of life satisfaction than their counterparts because they typically have fewer social contacts and a less active social life than their counterparts (Pollet et al., 2011). Since this is, presumably, already so before the first cohabitation, one may expect that more neurotic and introverted individuals gain more life satisfaction from the start of cohabitation than other persons. A partner’s presence and social network significantly increase opportunities for new social interaction and social and emotional support in a more familiar setting. Additionally, more neurotic and introverted individuals can expect social support from their partners without interacting with lots of different people. A partner may also increase a neurotic person’s life satisfaction more than an emotionally stable person because a companion may balance feelings, decrease worries, and provide more security. Vice versa, the loss of a partner, primarily through widowhood, is expected to lower life satisfaction more for neurotic and introverted individuals than for others. Even if cohabitation may have led to new social interactions, one may assume that most of the new social interactions of neurotic and introverted individuals were ex-partner’s contacts and are ‘taken’ by the ex-partner. Neurotic and introverted individuals may therefore cope socially less well with separation and widowhood than emotionally stable and extraverted individuals, who had a more extensive social network to start with. Separation and widowhood may, in addition, decrease the happiness of neurotic individuals because of the loss of the partner and their protective influence on worries and distress.

Thus, in contrast to the prediction from the Big Five theory (cf. H1), we expect from the social support perspective that the positive effect of the start of cohabitation on life satisfaction will be stronger (more positive) for more neurotic and introverted individuals than for others (H4). On the other hand, the negative effect of separation and widowhood on life satisfaction will be stronger (more negative) for more neurotic and introverted individuals than for others (H5). The latter prediction overlaps with the prediction from the Big Five theory (cf. H2), yet not with the prediction from the stress perspective, which only expected moderation by neuroticism (H3).

## Data, Measures, and Method

### Data

We use longitudinal panel data to test our hypotheses, in specific 28 (year-)waves from the German Socio-Economic Panel (SOEP; doi:10.5684/soep-core.v35). SOEP is one of the most extensive and longest-running household panel surveys worldwide, first conceived in 1984 and running up to 2020. It is based on national representative samples of individuals 16 years of age and older living in private households in Germany. These individuals are followed year-by-year within the panel. In addition, to ensure representativeness and tackle attrition, refreshment samples are added.^1^ “New” individuals become part of the SOEP population by being born into SOEP households or due to residential mobility. In total, every year about 30,000 respondents from 15,000 households are surveyed on a wide range of topics through face-to-face interviews and questionnaires. Further information on sampling and design can be obtained from Goebel et al., (2018).

Because information on the presence of a partner in the household is only available for waves 1991–2018, we restrict our analyses to this period. Our sample of analysis consists of 359,052 person-years from 35,413 respondents, an average of 10.1 included waves per person (the minimum is two and the maximum 28 waves; unbalanced panel). We selected individuals 18 years of age and older and did not impose an upper limit because of our interest in the widowhood effect. We furthermore excluded respondents with any missing or invalid responses on life satisfaction (6% of respondents) and partnership status (10%), and excluded respondents who were not addressed questions on personality traits (17% of all respondents) or did not answer these questions (less than 1%).^2^ In addition, since we are interested in the short-term, within one-year consequences of relationship transitions on life satisfaction (see method section), we selected respondents who participated in each wave (i.e., without gaps between year waves; 15% of respondents omitted).

### Measures

#### Life Satisfaction

The dependent variable life satisfaction is measured with the question “How satisfied are you with your life, all things considered?”, where responses were 0 (“completely dissatisfied”) to 10 (“completely satisfied”). This general measure of life satisfaction minimizes inaccuracies based on the personal prioritization of distinct life domains for happiness. It is frequently used in studies on relationship transitions and well-being (Roberson et al., 2018) and is also central in the abovementioned studies on personality and relationship transition effects (Anusic et al., 2014; Boyce et al., 2016; Yap et al., 2012).

#### Cohabitation, Separation, Widowhood

Our dynamic panel models estimate the effects of the start and end of cohabitation, through separation or widowhood, on life satisfaction. The start of cohabitation is measured as a change from not living with a partner in a household in year *t*-1 to living with a partner in a household in the subsequent year *t*, married or unmarried (self-reported; note that widowed and separated individuals may also start to cohabit). Cohabitation both includes heterosexual and gay couples. The end of cohabitation involves a change from living with a partner in year *t*-1 to not living with a partner in the subsequent wave *t*, irrespective of marital status. When respondents stated to be widowed in a year, yet not in the previous, this indicates a widowhood effect. Otherwise, the end of cohabitation involves separation. In total, we observed 4571 transitions into cohabitation during the panel (2010 for men and 2561 for women), 3434 transitions into separation (1452 for men and 1982 for women), and 1138 transitions into widowhood (326 for men and 812 for women; also see Table 1). We focus on the start and end of (married or unmarried) cohabitation rather than the timing of marriage and divorce since life satisfaction responds stronger to the presence or absence of a partner in the household than to changes in legal marital status (cf. Brown [2004] for cohabitation and marriage). We checked whether excluding persons who prior separated affected our findings, but it did not (see Appendix, Tables A.3-A.4).

**Table 1:** Descriptive statistics (person-years)

	Men		Women	
	Mean	SD	Mean	SD
Life satisfaction (0–10)	7.15	1.70	7.14	1.74
Cohabitation event (0–1)^a^	0.05 [N = 2010]		0.05 [N = 2561]	
Separation event (0–1)^b^	0.01 [N = 1452]		0.01 [N = 1982]	
Widowhood event (0–1)^b^	0.00 [N = 326]		0.00 [N = 812]	
Age (18–105)	48.26	16.96	48.23	17.16
Neuroticism (1–7)	3.61	1.03	4.10	1.07
Extraversion (1–7)	4.70	1.01	4.92	1.01
Conscientiousness (1–7)	5.78	0.84	5.92	0.78
Openness (1–7)	4.42	1.05	4.57	1.09
Agreeableness (1.33-7)	5.23	0.85	5.56	0.81
Observations	168,695		190,357	
Individuals	16,842		18,571	

^a^For years in which persons do not cohabit

^b^ For years in which persons cohabit

Unstandardized variables, minimum and maximum observed values between brackets

Source: SOEP 2018 (waves 1991–2018, unweighted); own calculations

#### Personality traits

Measures of Big Five personality traits are included in a limited number of SOEP waves (2005, 2009, 2012, 2013, and 2017; see Gerlitz & Schupp 2005). The Big-Five-Inventory-Short-Version (BIF-S) consists of 15 items, three items per trait, which allows collecting information on the respondent’s personality within a short amount of time. We measure neuroticism as the average value in a year of the items “I see myself as someone who worries a lot / somewhat nervous / deals well with stress”, where we reverse coded the item “deals well with stress” (response codes for all personality items are 1 “does not apply” to 7 “applies fully”). Extraversion is based on the average value of the items “I see myself as someone who is communicative / reserved / sociable”, where we reverse-coded the item “reserved”. Openness is based on the average value of the items “I see myself as someone who is original / values artistic experiences / lively imagination”. Agreeableness is based on the average value of the items “I see myself as someone who is sometimes too coarse with others / able to forgive / friendly with others”, where we reverse-coded the item “being coarse” (for a similar procedure, see Boyce et al., 2016; Yap et al., 2012). The BIF-S traits have a weaker internal consistency than long-version measures (Rammstedt et al., 2013). Still, Cronbach’s alpha values for the year-specific personality measures are acceptable given the restricted number of items (observed Cronbach’s alpha values are higher than 0.60, except for agreeableness [0.48]; cf. Donnellan & Lucas, 2008; Gerlitz & Schupp, 2005). Furthermore, high correlations between results obtained by the short and the long measure indicate that the BIF-S primarily reflects the structure of the BIF-25 and is thus an appropriate measure (Donnelann & Lucas, 2008; Gerlitz & Schupp 2005).

Since personality is measured in only a few survey years, we observe within-individual time variation in personality (30% of total variation; the rest [70%] is the between-individual variation; cf. Appendix, Tables A.5-A.6). This variation is larger than if we had personality measures each year (cf. Atherton et al., 2021). To increase the reliability of the personality measures, we averaged the year-specific personality measures for respondents with more than one measurement during the panel (76% of respondents). It is important to note that we did not observe effects of cohabitation, separation, or widowhood on (changes in) personality traits so that our aggregation procedure is justified (cf. Appendix, Tables A.5-A.6). Other studies neither revealed substantial effects of relationship transitions on personality (cf. Bleidorn et al., 2018; Specht et al., 2011; Wagner et al., 2015).

#### Controls

Because our fixed effects regressions control for any time-constant individual-level variable by design (see below), we only use age as a control variable in the analyses. Age is important to control as it associates with relationship transitions and life satisfaction (Gerstorf et al., 2008).^3^

Table 1 list the means of the variables of interest by gender. Note that variables are standardized (mean-centered) in our fixed effects regressions.

### Method

We use fixed effects, linear regression models to estimate relationship transition effects on life satisfaction and moderation of these effects by personality traits (cf. Allison, 2009; Amato, 2010). The models estimate the effects of intra-individual change in an independent variable on intra-individual change in a dependent variable by fixing all respondents, that is, as if a dummy variable is included for each respondent. These dynamic panel models approximate causation better than cross-section models as they focus on change. Furthermore, the models keep all time-constant variables constant by design and thus cancel out time-constant observed and unobserved variables that may disturb the relationships of interest (factors that may be both causes of relationship transitions and life satisfaction). Earlier dynamic analyses of relationship transitions and life satisfaction and well-being provided more support for relationship transition effects on life satisfaction than reverse causal effects (i.e., self-selection; see review studies of Amato [2010] on divorce and well-being, Johnson and Wu [2002] and DeMaris [2018] on marriage and well-being, and Luhman et al. [2012] on multiple life events and well-being). We also observed this in our analyses as life satisfaction peaks in the year cohabitation, separation, or widowhood occurred (cf. Appendix, Figure A.1). Note that within our fixed effects regressions, the main effects of personality traits are not estimated because these are measured as aggregated means per respondent. However, the interactive effects of personality with relationship transitions are estimated (cf. Giesselmann & Schmidt-Catran, 2020).

Our analytical strategy involves three important decisions. Firstly, we estimate the effects of the start and the end of cohabitation for distinct subsamples (cf. Yap et al., 2012). The analyses of the effect of cohabitation start on life satisfaction are based on a subsample of individuals who do not cohabit. The analyses of the effect of separation and widowhood on life satisfaction are based on a subsample of individuals who cohabit. We split our analyses since relationship transitions have asymmetrical effects on life satisfaction. The start of (married) cohabitation, for example, has a smaller effect on life satisfaction than widowhood (cf. Holmes & Rahe, 1967; Lucas et al., 2003). This asymmetry cannot be modeled in a pooled design. Secondly, as already mentioned, we focus on the short-term, within one-year effects of relationship transitions on life satisfaction. We do this because these effects are most substantial within this period. After that, life satisfaction fairly quickly returns to pre-event levels, although this occurs more slowly after widowhood (Brickman et al., 1978; Lucas et al., 2003; Luhmann et al., 2012; Yap et al., 2012). The short-term nature of effects is also documented in robustness analyses, where we observed a peak in life satisfaction in the event year (Appendix, Figure A.1). Thirdly, we split the analyses by gender. We do this because men and women differ in the well-being consequences of cohabitation, separation, and widowhood (Stronge et al., 2019; Terhell et al., 2004). For men and women pooled analyses are displayed in the Appendix (Tables A.7-A.8). In these additional analyses, we also estimated gender differences in covariate estimates. The script used for our analyses can be found in the Open Science Framework (OSF; https://osf.io/vpr4y/).

## Findings

Our fixed effects regressions show that the start of cohabitation is associated with an increase in life satisfaction among men and women (Table 2). For men, cohabitation raises life satisfaction by 0.37 on the 0–10 scale, which is a ([6.76 + 0.37]/6.76=] 5% increase compared to life satisfaction in non-cohabitation (cf. Model 1). For women, cohabitation raises life satisfaction by 0.39, a 6% increase compared to non-cohabitation (cf. Model 3; effects for men and women do not statistically differ; cf. Appendix, Table A.7). Although the effect of the start of cohabitation on life satisfaction seems small, variation in people’s life satisfaction is generally low, as most people are relatively satisfied with life (cf. Gustavson et al., 2016). As a share of the total within-individual variation in life satisfaction, the short-term change in satisfaction with cohabitation is substantial: (0.37/1.49=) 25% for men and (0.39/1.61=) 24% for women.

**Table 2:** Fixed effects regression of life satisfaction: the effect of cohabitation start^a^ by personality traits

	Men				Women			
	(1)		(2)		(3)		(4)	
Age	-0.367^**^	(0.027)	-0.367^**^	(0.027)	-0.209^**^	(0.023)	-0.208^**^	(0.023)
Cohabitation	0.365^**^	(0.034)	0.370^**^	(0.038)	0.386^**^	(0.031)	0.368^**^	(0.034)
Cohab. x neuroticism^b^			0.004	(0.037)			0.051	(0.033)
Cohab. x extraversion			-0.054	(0.037)			0.060	(0.034)
Cohab. x conscientiousness			0.027	(0.034)			0.036	(0.033)
Cohab. x openness			-0.012	(0.039)			-0.043	(0.034)
Cohab. x agreeableness			-0.026	(0.036)			0.015	(0.034)
Constant	6.763^**^	(0.018)	6.763^**^	(0.018)	6.913^**^	(0.006)	6.913^**^	(0.006)
Observations	42,522		42,522		56,663		56,663	
Individuals	6270		6270		7988		7988	
Log likelihood	-67065.1		-67062.7		-91725.5		-91721.7	

Standardized variables; standard errors in parentheses;

^*^p < 0.05, ^**^p < 0.01

^a^ For years in which persons do not cohabit

^b^ Main effects of personality traits not estimated since time-constant

Source: SOEP 2018 (waves 1991–2018, unweighted); own calculations

Notwithstanding, Table 2 shows that the Big Five personality traits do not moderate the effect of the start of cohabitation on life satisfaction, neither for men nor women.^4^ This is neither the case when focusing on individuals with high or low values on personality traits, for example, highly neurotic or introverted individuals. We only find that the cohabitation effect is significantly larger for men who belong to the highest quantile of conscientiousness (Appendix, Table A.9). These findings regarding cohabitation, personality, and life satisfaction reject the predictions from the Big Five theory (H1) and the social support perspective (H4).

Table 3 displays that the end of cohabitation, through separation or widowhood, has larger effects on life satisfaction than the cohabitation start. This finding underlines the asymmetry in relationship transition effects. Among men, separation decreases life satisfaction by 0.74, which is a 10% decrease compared to cohabitation (cf. Model 1). Among women, the separation effect is -0.52, a 7% decrease in life satisfaction (Model 4; gender difference in separation effect is significant; cf. Appendix, Table A.8). Widowhood decreases life satisfaction even more. Among men, widowhood decreases life satisfaction by 1.27, an 18% decrease compared to cohabitation (Model 1). Among women, widowhood reduces life satisfaction by 1.37, a 19% decrease compared to cohabitation (Model 4; gender difference in widowhood effect is not significant; cf. Appendix, Table A.8).

**Table 3:** Fixed effects regression of life satisfaction: the effect of separation and widowhood^a^ by personality traits

	Men						Women					
	(1)		(2)		(3)		(4)		(5)		(6)	
Age	-0.275^**^	(0.011)	-0.275^**^	(0.011)	-0.275^**^	(0.011)	-0.280^**^	(0.012)	-0.280^**^	(0.012)	-0.280^**^	(0.012)
Separation	-0.744^**^	(0.036)	-0.773^**^	(0.038)	-0.743^**^	(0.036)	-0.517^**^	(0.032)	-0.525^**^	(0.035)	-0.517^**^	(0.032)
Widowhood	-1.273^**^	(0.070)	-1.274^**^	(0.070)	-1.333^**^	(0.078)	-1.368^**^	(0.046)	-1.368^**^	(0.046)	-1.205^**^	(0.054)
Sep. x neuroticism^b^			-0.109^**^	(0.037)					-0.025	(0.033)		
Sep. x extraversion			0.001	(0.040)					-0.001	(0.035)		
Sep. x conscientiousness			0.074	(0.039)					0.067	(0.035)		
Sep. x openness			0.012	(0.039)					0.062	(0.034)		
Sep. x agreeableness			-0.046	(0.037)					-0.030	(0.034)		
Wid. x neuroticism					-0.065	(0.083)					-0.196^**^	(0.049)
Wid. x extraversion					-0.002	(0.087)					0.112	(0.060)
Wid. x conscientiousness					0.088	(0.079)					0.021	(0.052)
Wid. x openness					-0.124	(0.079)					-0.014	(0.053)
Wid. x agreeableness					0.032	(0.075)					-0.260^**^	(0.053)
Constant	7.265^**^	(0.004)	7.265^**^	(0.004)	7.265^**^	(0.004)	7.252^**^	(0.003)	7.252^**^	(0.003)	7.252^**^	(0.003)
Observations	126,173		126,173		126,173		133,694		133,694		133,694	
Individuals	12,796		12,796		12,796		13,814		13,814		13,814	
Log likelihood	-193,321		-193,313		-193,318		-207,912		-207,906		-207,886	

Standardized variables; standard errors in parentheses;

^*^p < 0.05, ^**^p < 0.01

^a^ For years in which persons cohabit

^b^ Main effects of personality traits not estimated since time-constant

Source: SOEP 2018 (waves 1991–2018, unweighted); own calculations

Contrary to cohabitation, personality traits moderate separation and widowhood effects on life satisfaction. For men, neuroticism increases the negative effect of separation (Model 2, Table 3); for women, neuroticism and agreeableness increase the negative effect of widowhood (Model 6). Extraversion, conscientiousness, and openness do not have (linear) moderating effects. Yet, women who score low on conscientiousness (quantile 1) experience more negative consequences of separation than other women, and women who score high on openness (quantile 5) experience less negative consequences of separation (Appendix, Table A.9). Nevertheless, the few significant interaction effects overall indicate that the moderation of separation and widowhood effects by personality traits is modest. The size of the interaction effects also evidences this. For men, a one standard deviation increase in neuroticism ‘only’ decreases life satisfaction after separation with an extra 2% points (12% instead of 10%; computed from Model 2, Table 2; see Appendix, Table A.9). For women, these added effects are 2% points for neuroticism and widowhood and 3% points for agreeableness and widowhood. An exception to this pattern of modest personality interactions is the widowhood effect for highly neurotic and low-agreeable women. For highly neurotic women (quantile 5 of neuroticism), the negative consequences of widowhood on life satisfaction are substantially stronger than for other women (about 6% points more). Conversely, for women scoring low on agreeableness (quantile 1), the negative consequences of widowhood are substantially weaker than for other women (about 7% points less; Appendix, Table A.9).^5^

The (somewhat) stronger effects of separation and widowhood for more neurotic individuals align with the prediction from the stress perspective (H3). The predictions from the Big Five theory (H2) and social support perspective (H5), on the other hand, are partially rejected as extraversion does not moderate separation and widowhood effects.

## Conclusions and Discussion

Our study investigated the moderating influence of personality traits, particularly of neuroticism and extraversion, on the effects of cohabitation, separation, and widowhood on life satisfaction. For that purpose, we employed 28 waves of the German Socio-Economic Panel 1991–2018 and estimated fixed effects regression models of intra-individual changes in life satisfaction. We observed that the start of (married or unmarried) cohabitation increases short-term life satisfaction and the end of cohabitation, through separation or widowhood, decreases short-term life satisfaction. However, these relationship transition effects are only to a limited extent moderated by personality traits. More neurotic men display a more negative effect of separation, and more neurotic and more agreeable women show a more negative effect of widowhood than their counterparts. These moderation effects are small, except for the widowhood effect for women scoring high on neuroticism and low on agreeableness. Furthermore, the other three Big Five traits (extraversion, conscientiousness, and openness) do not moderate the influence of relationship transitions on life satisfaction, and the effect of cohabitation start on life satisfaction is not at all moderated by Big Five traits.

Our finding of a limited moderation of relationship transition effects on life satisfaction by personality traits is in line with prior studies. Boyce et al., (2016) observed an inconsistent pattern of moderation of marriage effects by personality traits for Germany. Pai & Carr (2010) observed only a weak interaction of widowhood and personality traits on depressive symptoms for the U.K. In addition, Yap et al., (2012) for the U.K. and Anusic et al., (2014) for Australia did not find moderating effects of personality regarding the effects of marriage and widowhood on life satisfaction. We improved upon these studies by using a larger panel data set with a greater number of transitions, fixed effects models, and looking at the moment of cohabitation and separation rather than legal marriage and divorce. Yet, we still hardly found moderating effects of personality.

However, a limitation of our study is that we compared life satisfaction from year to year. The strongest effects of cohabitation, separation, and widowhood may come more immediately, and personality traits may then join in. Future studies may want to investigate this by focusing on more short-term changes in life satisfaction, for example, by using monthly well-being thermometers, as in surveys on the consequences of the COVID-19 pandemic. Another limitation of our study is that we could not measure the underlying mechanisms of the moderation effects. From the social support perspective, we expected that the end of cohabitation poses more adverse effects for more neurotic people due to weaker social support. From the stress perspective, we expected this more adverse effect due to accumulated stress. A more direct test would be to examine whether relationship transition effects differ for individuals with varying levels of stress and social support (cf. Amato, 2010) and whether this variation can account for moderation by personality (cf. Pai & Carr, 2010). A final limitation is that we focused our analyses on life satisfaction and cannot test relationship transition effects and its moderation by personality for other dimensions of subjective well-being, specifically positive and negative affect. However, because the effects of life events are weaker and more short-term on affective components than on the cognitive component of well-being (i.e., life satisfaction; cf. Luhmann et al., 2012), we think moderation of affective dimensions by personality will be even weaker (cf. Anusic et al., 2014). Still, this assumption needs further testing.

Substantively, our findings support the predictions from the stress perspective most. Although the moderating influence is generally small, more neurotic individuals experience more negative effects of separation (men) and widowhood (women) than others. This may be explained by the stress caused by separation and widowhood, for example, the multiple changes these events cause in people’s lives (Amato, 2010). Neurotic individuals may handle this stress less well than others, which leads to disproportionate decreases in life satisfaction. Alternatively, the more negative effect of separation and widowhood for neurotic individuals may be attributed to the loss of social support upon separation and widowhood. Yet, this social support explanation is less supported in our study since we do not observe a disproportionate increase in life satisfaction for neurotic individuals with the start of cohabitation. Furthermore, we do not find that extraversion moderates relationship transition effects, something the social support perspective expected.

Our results have several practical implications. Knowledge of the consequences of relationship transitions for different groups of individuals is fundamental to better support vulnerable groups (e.g., highly neurotic) in the stage of primary prevention. Here the aim is to minimize separation risks with interventions that increase couples’ capabilities of dealing with conflict and cultivate the relationship’s positive dimensions (e.g., Prevention and Relationship Enhancement Program [PREP]; Renick et al., 1992). Yet, in many cases, partnership dissolution is inevitable, and secondary prevention strategies must be used to minimize the aversiveness of the event. Community group-based intervention programs have, in this respect, shown to be effective in supporting the adjustment after partnership dissolution (Vukalovich & Caltabiano, 2008). Increasing the availability of such support services and awareness about specifically vulnerable groups could help vulnerable groups better cope with the situation. In addition, group-specific interventions may be helpful. Neurotic individuals in relationship transitions could be supported with interventions aimed at reducing levels of neuroticism, for example, mindfulness-based cognitive therapy (Armstrong & Rimes, 2016). Widowed individuals could be supported by intervention programs focusing on physical activity (Williams et al., 2021). Although more research is needed to establish robust findings, such interventions appear to be a promising avenue to decrease the adverse relationship transition effects on life satisfaction.

## Electronic supplementary material

Below is the link to the electronic supplementary material.

Supplementary Material 1

## Footnotes

1 Attrition is similar to other large household panel surveys (Watson & Wooden, 2011).

2 When including respondents with missing information on personality, we found the same effects of relationship transitions on life satisfaction (Appendix, Tables A.1-A.2).

3 Unfortunately, we miss good longitudinal measures of social contacts in our data to inspect whether changes in social contacts drive changes in life satisfaction for more neurotic, introverted, and other individuals. SOEP only includes a survey question on the number of (close) friends for the years 2003, 2008, 2013, 2015, 2017, and 2018. A control for the number of friends prior to cohabitation or inclusion of a dynamic measure of friends is, therefore, not an option. Note that for each wave for which we have valid information on the number of friends, we observed that more neurotic and introverted individuals report a significantly lower number of (close) friends than their counterparts, in line with the assumption of the social support perspective.

4 For women, the interactive effect of cohabitation by extraversion is significantly larger than for men (cf. Appendix Table A.7). Yet, the interactive effect for women separately is still insignificant (cf. Table 2, Model 4).

5 We checked whether our central findings regarding the moderation of relationship effects by personality were robust for distinct population groups, as for migration status and sexual orientation. We observed the same findings when excluding the minority groups of these group variables (respectively, migrants and gay and lesbian couples; cf. Appendix Tables, A.10-A.13). Separate analyses for these minority groups was not feasible due to low sample sizes.
