# Supplementary material for: Do Personality Traits Moderate the Effects of Cohabitation, Separation, and Widowhood on Life Satisfaction? A Longitudinal Test for Germany
Source: J Happiness Stud. 2022 Nov 2;24(1):141–57. doi: 10.1007/s10902-022-00573-8 (PMC9628624; doi:10.1007/s10902-022-00573-8)
Supplement: Supplementary file 1 — Supplementary Material 1 [file 10902_2022_573_MOESM1_ESM.docx]

**ONLINE APPENDIX - Do personality traits moderate the effects of cohabitation, separation, and widowhood on life satisfaction? A longitudinal test for Germany**

Table A.1. Fixed effects regression of life satisfaction: the effect of cohabitation start^a^, including respondents with missing personality measures

|  | Men  (1) |  | Women  (2) |  |
| --- | --- | --- | --- | --- |
| Age | -0.417^**^ | (0.027) | -0.276^**^ | (0.024) |
| Cohabitation | 0.332^**^ | (0.032) | 0.381^**^ | (0.030) |
| Constant | 6.711^**^ | (0.019) | 6.886^**^ | (0.005) |
| Observations | 51344 |  | 68151 |  |
| Individuals | 8581 |  | 10737 |  |
| Log likelihood | -81222.5 |  | -110645.8 |  |

Standardized variables; standard errors in parentheses

^*^ *p* < 0.05, ^**^ *p* < 0.01

^a^ For years in which individuals do not cohabit

Source: SOEP 2018 (waves 1991-2018, unweighted); own calculations

Table A.2. Fixed effects regression of life satisfaction: the effects of separation and widowhood^a^, including respondents with missing personality measures

|  | Men  (1) |  | Women  (3) |  |
| --- | --- | --- | --- | --- |
| Age | -0.309^**^ | (0.012) | -0.312^**^ | (0.012) |
| Separation | -0.722^**^ | (0.034) | -0.502^**^ | (0.030) |
| Widowhood | -1.169^**^ | (0.065) | -1.335^**^ | (0.044) |
| Constant | 7.229^**^ | (0.004) | 7.224^**^ | (0.003) |
| Observations | 146364 |  | 152799 |  |
| Individuals | 17268 |  | 18117 |  |
| Log likelihood | -224994.6 |  | -238101.4 |  |

Standardized variables; standard errors in parentheses

^*^ *p* < 0.05, ^**^ *p* < 0.01

^a^ For years in which individuals cohabit

^b^ Main effects of personality traits not estimated since time-constant.

Source: SOEP 2018 (waves 1991-2018, unweighted); own calculations

Table A.3. Fixed effects regression of life satisfaction: the effect of cohabitation start^a^ by personality traits, excluding those who prior separated

|  | Men  (1) |  | Women  (2) |  |
| --- | --- | --- | --- | --- |
| Age | -0.475^**^ | (0.031) | -0.330^**^ | (0.028) |
| Cohabitation | 0.329^**^ | (0.044) | 0.328^**^ | (0.039) |
| Cohab. x neuroticism^b^ | -0.023 | (0.041) | 0.053 | (0.038) |
| Cohab. x extraversion | -0.055 | (0.041) | 0.048 | (0.038) |
| Cohab. x conscientiousness | 0.007 | (0.037) | 0.037 | (0.038) |
| Cohab. x openness | 0.006 | (0.045) | -0.057 | (0.039) |
| Cohab. x agreeableness | -0.015 | (0.040) | -0.027 | (0.039) |
| Constant | 6.704^**^ | (0.027) | 6.987^**^ | (0.008) |
| Observations | 35051 |  | 42733 |  |
| Individuals | 5236 |  | 6353 |  |
| Log likelihood | -54866.182 |  | -67967.223 |  |

Standardized variables; standard errors in parentheses

^*^ *p* < 0.05, ^**^ *p* < 0.01

^a^ For years in which individuals do not cohabit

^b^ Main effects of personality traits not estimated since time-constant.

Source: SOEP 2018 (waves 1991-2018, unweighted); own calculations

Table A.4. Fixed effects regression of life satisfaction: the effects of separation and widowhood^a^ by personality traits, excluding those who prior separated

|  | Men  (1) |  | (2) |  | Women  (3) |  | (4) |  |
| --- | --- | --- | --- | --- | --- | --- | --- | --- |
| Age | -0.285^**^ | (0.012) | -0.285^**^ | (0.012) | -0.285^**^ | (0.012) | -0.285^**^ | (0.012) |
| Separation | -0.783^**^ | (0.041) | -0.739^**^ | (0.038) | -0.482^**^ | (0.038) | -0.489^**^ | (0.034) |
| Widowhood | -1.268^**^ | (0.071) | -1.325^**^ | (0.078) | -1.356^**^ | (0.046) | -1.185^**^ | (0.054) |
| Sep. x neurotiscm^b^ | -0.158^**^ | (0.040) |  |  | -0.047 | (0.035) |  |  |
| Sep. x extraversion | -0.014 | (0.042) |  |  | -0.027 | (0.038) |  |  |
| Sep. x conscientiousness | 0.067 | (0.042) |  |  | 0.066 | (0.038) |  |  |
| Sep. x openness | -0.021 | (0.042) |  |  | 0.051 | (0.036) |  |  |
| Sep. x agreeableness | -0.062 | (0.040) |  |  | -0.022 | (0.036) |  |  |
| Wid. x neuroticism |  |  | -0.068 | (0.083) |  |  | -0.197^**^ | (0.049) |
| Wid. x extraversion |  |  | 0.001 | (0.088) |  |  | 0.117 | (0.060) |
| Wid. x conscientiousness |  |  | 0.082 | (0.080) |  |  | 0.034 | (0.052) |
| Wid. x openness |  |  | -0.118 | (0.080) |  |  | -0.006 | (0.053) |
| Wid. x agreeableness |  |  | 0.035 | (0.075) |  |  | -0.269^**^ | (0.053) |
| Constant | 7.271^**^ | (0.004) | 7.271^**^ | (0.004) | 7.259^**^ | (0.003) | 7.259^**^ | (0.003) |
| Observations | 121635 |  | 121635 |  | 128764 |  | 128764 |  |
| Individuals | 12438 |  | 12438 |  | 13419 |  | 13419 |  |
| Log likelihood | -185728 |  | -185737 |  | -199627 |  | -199603 |  |

Standardized variables; standard errors in parentheses

^*^ *p* < 0.05, ^**^ *p* < 0.01

^a^ For years in which individuals cohabit

^b^ Main effects of personality traits not estimated since time-constant.

Source: SOEP 2018 (waves 1991-2018, unweighted); own calculations

Table A.5. Fixed effects regression of the effect of cohabitation start^a^ on personality traits

|  | (1) | (2) | (3) | (4) | (5) |
| --- | --- | --- | --- | --- | --- |
|  | neuroticism | extravers. | conscient. | openness | agreeable |
| Age | -0.011^**^ | -0.006^**^ | -0.009^**^ | -0.007^**^ | -0.009^**^ |
|  | (0.002) | (0.002) | (0.002) | (0.002) | (0.002) |
| Cohabitation | 0.017 | -0.072 | 0.072 | -0.174^**^ | -0.005 |
|  | (0.057) | (0.050) | (0.046) | (0.055) | (0.047) |
| Constant | 4.309^**^ | 5.137^**^ | 6.007^**^ | 4.915^**^ | 5.833^**^ |
|  | (0.099) | (0.087) | (0.081) | (0.096) | (0.082) |
| Observations | 20001 | 19992 | 19940 | 19912 | 19996 |
| Individuals | 12470 | 12472 | 12463 | 12469 | 12472 |
| % Between-individ. var. | 69% | 71% | 71% | 68% | 66% |
| Log Likelihood | -14627.0 | -11903.3 | -10317.9 | -13711.2 | -10843.5 |

Standard errors in parentheses

^*^ *p* < 0.05, ^**^ *p* < 0.01

^a^ For years in which individuals do not cohabit

Source: SOEP 2018 (waves 1991-2018, unweighted); own calculations

Table A.6. Fixed effects regression of the effects of separation and widowhood^a^ on personality traits

|  | (1) | (2) | (3) | (4) | (5) |
| --- | --- | --- | --- | --- | --- |
|  | neuroticism | extravers. | conscient. | openness | agreeable |
| Age | -0.014^**^ | -0.006^**^ | -0.015^**^ | -0.000 | -0.008^**^ |
|  | (0.001) | (0.001) | (0.001) | (0.001) | (0.001) |
| Separation | 0.044 | 0.031 | -0.043 | 0.123^*^ | 0.078 |
|  | (0.057) | (0.050) | (0.045) | (0.056) | (0.050) |
| Widowhood | 0.111 | -0.048 | 0.085 | -0.211^*^ | 0.114 |
|  | (0.088) | (0.076) | (0.069) | (0.084) | (0.075) |
| Constant | 4.569^**^ | 5.140^**^ | 6.671^**^ | 4.549^**^ | 5.792^**^ |
|  | (0.061) | (0.052) | (0.047) | (0.059) | (0.052) |
| Observations | 48163 | 48151 | 48102 | 47982 | 48139 |
| Individuals | 25287 | 25286 | 25280 | 25280 | 25283 |
| % Between-individ. var. | 70% | 71% | 67% | 69% | 65% |
| Log Likelihood | -38398.1 | -31249.8 | -26361.6 | -36492.5 | -30999.0 |

Standard errors in parentheses

^*^ *p* < 0.05, ^**^ *p* < 0.01

^a^ For years in which individuals cohabit

Source: SOEP 2018 (waves 1991-2018, unweighted); own calculations

Table A.7. Fixed effects regression of cohabitation start and personality on life satisfaction: for men and women pooled analyses with interactions by gender^a^

| Age | -0.367^**^ | (0.028) |
| --- | --- | --- |
| Cohabitation | 0.370^**^ | (0.039) |
| Cohabitation # neuroticism | 0.004 | (0.037) |
| Cohabitation # extraversion | -0.054 | (0.037) |
| Cohabitation # conscientiousness | 0.027 | (0.035) |
| Cohabitation # openness | -0.012 | (0.040) |
| Cohabitation # agreeableness | -0.026 | (0.037) |
| Age # woman | 0.158^**^ | (0.036) |
| Cohabitation # woman | -0.002 | (0.051) |
| Cohabitation # neuroticism # woman | 0.047 | (0.050) |
| Cohabitation # extraversion # woman | 0.114^*^ | (0.050) |
| Cohabitation # conscientiousness # woman | 0.009 | (0.048) |
| Cohabitation # openness # woman | -0.030 | (0.052) |
| Cohabitation # agreeableness # woman | 0.041 | (0.050) |
| Constant | 7.886^**^ | (0.070) |
| Observations | 99185 |  |
| Individuals | 14258 |  |
| Log Likelihood | -158826.2 |  |

Standardized variables; standard errors in parentheses

^*^ *p* < 0.05, ^**^ *p* < 0.01

^a^ For years in which individuals do not cohabit

Source: SOEP 2018 (waves 1991-2018, unweighted); own calculations

Table A.8. Fixed effects regression of separation, widowhood, and personality on life satisfaction: for men and women pooled analyses with interactions by gender^a^

|  | (1) |  | (2) |  |
| --- | --- | --- | --- | --- |
| Age | -0.275^**^ | (0.012) | -0.275^**^ | (0.012) |
| Separation | -0.773^**^ | (0.039) | -0.743^**^ | (0.036) |
| Widowhood | -1.274^**^ | (0.071) | -1.333^**^ | (0.079) |
| Sep. # neuroticism | -0.109^**^ | (0.038) |  |  |
| Sep. # extraversion | 0.001 | (0.040) |  |  |
| Sep. # conscientiousness | 0.074 | (0.040) |  |  |
| Sep. # openness | 0.012 | (0.039) |  |  |
| Sep. # agreeableness | -0.046 | (0.037) |  |  |
| Wid. # neuroticism |  |  | -0.065 | (0.084) |
| Wid. # extraversion |  |  | -0.002 | (0.088) |
| Wid. # conscientiousness |  |  | 0.088 | (0.080) |
| Wid. # openness |  |  | -0.124 | (0.080) |
| Wid. # agreeableness |  |  | 0.032 | (0.076) |
| Age # woman | -0.005 | (0.016) | -0.005 | (0.016) |
| Sep. # woman | 0.248^**^ | (0.052) | 0.227^**^ | (0.048) |
| Wid. # woman | -0.094 | (0.085) | 0.128 | (0.095) |
| Sep. # neuroticism # woman | 0.085 | (0.050) |  |  |
| Sep. # extraversion # woman | -0.002 | (0.053) |  |  |
| Sep. # conscientiousness # woman | -0.008 | (0.053) |  |  |
| Sep. # openness # woman | 0.050 | (0.052) |  |  |
| Sep. # agreeableness # woman | 0.016 | (0.050) |  |  |
| Wid. # neuroticism # woman |  |  | -0.131 | (0.097) |
| Wid. # extraversion # woman |  |  | 0.115 | (0.106) |
| Wid. # conscientiousness # woman |  |  | -0.067 | (0.095) |
| Wid. # openness # woman |  |  | 0.110 | (0.095) |
| Wid. # agreeableness # woman |  |  | -0.291^**^ | (0.092) |
| Constant | 7.258^**^ | (0.003) | 7.258^**^ | (0.003) |
| Observations | 259867 |  | 259867 |  |
| Individuals | 26610 |  | 26610 |  |
| Log Likelihood | -401253.3 |  | -401237.3 |  |

Standardized variables; standard errors in parentheses

^*^ *p* < 0.05, ^**^ *p* < 0.01

^a^ For years in which individuals cohabit

Source: SOEP 2018 (waves 1991-2018, unweighted); own calculations

Table A.9. Estimated change in life satisfaction with cohabitation, separation, and widowhood for quantiles of personality traits^a^

|  |  | Quantiles of personality | | |  |  |
| --- | --- | --- | --- | --- | --- | --- |
|  |  | (1) | (2) | (3) | (4) | (5) |
| A. Cohabitation effect | |  |  |  |  |  |
| Men | Neuroticism | 5.4% | 3.6% | 6.8% | 5.1% | 6.9% |
|  | Extraversion | 6.9% | 4.2% | 5.9% | 5.3% | 4.8% |
|  | Conscientiousness | 4.8% | 5.7% | 4.6% | 5.2% | **8.9%** |
|  | Openness | 6.5% | 4.7% | 4.8% | 6.3% | 4.8% |
|  | Agreeableness | 6.1% | 5.2% | 4.4% | 5.8% | 5.3% |
| Women | Neuroticism | 5.4% | 5.3% | 4.4% | 5.9% | 6.5% |
|  | Extraversion | 5.6% | 4.2% | 4.8% | 4.9% | 7.1% |
|  | Conscientiousness | 7.0% | **2.7%** | **4.4%** | 6.1% | 8.7% |
|  | Openness | 6.2% | 4.7% | 5.6% | 5.9% | 5.5% |
|  | Agreeableness | 6.5% | 5.5% | 5.0% | **3.6%** | 7.4% |
|  |  |  |  |  |  |  |
| B. Separation effect | |  |  |  |  |  |
| Men | Neuroticism | -9.4% | -9.1% | -11.7% | -10.6% | -11.6% |
|  | Extraversion | -10.6% | -10.6% | -11.0% | -9.1% | -10.0% |
|  | Conscientiousness | -12.9% | **-9.5%** | -10.2% | **-8.3%** | -10.3% |
|  | Openness | -11.4% | -10.6% | -9.7% | -9.7% | -9.9% |
|  | Agreeableness | -10.6% | **-7.4%** | **-6.9%** | **-13.9%** | -9.7% |
| Women | Neuroticism | -6.3% | -5.5% | **-9.4%** | -5.2% | -8.6% |
|  | Extraversion | -8.3% | -8.0% | -6.6% | -5.9% | -7.3% |
|  | Conscientiousness | -10.6% | **-5.3%** | **-7.2%** | **-6.1%** | **-7.1%** |
|  | Openness | -8.6% | -8.2% | -7.6% | -6.3% | **-5.8%** |
|  | Agreeableness | -10.6% | **-6.0%** | -11.2% | -9.6% | -10.1% |
|  |  |  |  |  |  |  |
| C. Widowhood effect | |  |  |  |  |  |
| Men | Neuroticism | -16.9% | -16.1% | -16.3% | -19.0% | -21.5% |
|  | Extraversion | -18.4% | -13.1% | -18.9% | -19.6% | -17.1% |
|  | Conscientiousness | -18.2% | -18.3% | -21.1% | -13.4% | -17.1% |
|  | Openness | -16.5% | -15.0% | -19.5% | -20.0% | -17.4% |
|  | Agreeableness | -18.0% | -15.0% | -21.9% | -18.6% | -14.5% |
| Women | Neuroticism | -17.0% | -13.8% | -18.0% | -18.1% | **-23.5%** |
|  | Extraversion | -22.9% | **-16.8%** | -19.8% | **-15.0%** | -19.5% |
|  | Conscientiousness | -18.8% | -20.4% | -19.4% | -16.8% | -19.5% |
|  | Openness | -20.0% | -17.6% | -18.5% | -16.4% | -21.8% |
|  | Agreeableness | -12.1% | -15.8% | **-18.5%** | **-21.0%** | **-21.0%** |

^a^ Estimated from fixed effects models of life satisfaction modeling the effects of cohabitation, separation, and widowhood interacted by quintiles of personality traits (models control for age). Numbers in bold indicate a statistically significant difference in the estimated change in life satisfaction compared to the first quintile (reference category in models; p<0.05).

Source: SOEP 2018 (waves 1991-2018, unweighted); own calculations

Table A.10. Fixed effects regression of life satisfaction: the effect of cohabitation start^a^ by personality traits, excluding those born outside Germany and those who immigrated after 1950

|  | Men  (1) |  | Women  (2) |  |
| --- | --- | --- | --- | --- |
| Age | -0.373^**^ | (0.028) | -0.202^**^ | (0.024) |
| Cohabitation | 0.372^**^ | (0.040) | 0.351^**^ | (0.036) |
| Cohab. x neuroticism^b^ | -0.013 | (0.038) | 0.054 | (0.034) |
| Cohab. x extraversion | -0.047 | (0.038) | 0.081^*^ | (0.035) |
| Cohab. x conscientiousness | 0.020 | (0.035) | 0.037 | (0.035) |
| Cohab. x openness | -0.006 | (0.041) | -0.050 | (0.035) |
| Cohab. x agreeableness | -0.037 | (0.037) | 0.019 | (0.036) |
| Constant | 6.741^**^ | (0.019) | 6.917^**^ | (0.006) |
| Observations | 39574 |  | 51872 |  |
| Individuals | 5752 |  | 7164 |  |
| Log likelihood | -62500.4 |  | -83693.4 |  |

Standardized variables; standard errors in parentheses

^*^ *p* < 0.05, ^**^ *p* < 0.01

^a^ For years in which individuals do not cohabit

^b^ Main effects of personality traits not estimated since time-constant.

Source: SOEP 2018 (waves 1991-2018, unweighted); own calculations

Table A.11. Fixed effects regression of life satisfaction: the effects of separation and widowhood^a^ by personality traits, excluding those born outside Germany and those who immigrated after 1950

|  | Men  (1) |  | (2) |  | Women  (3) |  | (4) |  |
| --- | --- | --- | --- | --- | --- | --- | --- | --- |
| Age | -0.261^**^ | (0.012) | -0.261^**^ | (0.012) | -0.263^**^ | (0.012) | -0.263^**^ | (0.012) |
| Separation | -0.773^**^ | (0.040) | -0.749^**^ | (0.037) | -0.514^**^ | (0.037) | -0.521^**^ | (0.034) |
| Widowhood | -1.323^**^ | (0.072) | -1.373^**^ | (0.080) | -1.376^**^ | (0.048) | -1.218^**^ | (0.057) |
| Sep. x neurotiscm^b^ | -0.082^*^ | (0.039) |  |  | -0.056 | (0.035) |  |  |
| Sep. x extraversion | -0.037 | (0.041) |  |  | -0.034 | (0.037) |  |  |
| Sep. x conscientiousness | 0.088^*^ | (0.041) |  |  | 0.059 | (0.037) |  |  |
| Sep. x openness | 0.029 | (0.041) |  |  | 0.081^*^ | (0.037) |  |  |
| Sep. x agreeableness | -0.050 | (0.038) |  |  | -0.043 | (0.037) |  |  |
| Wid. x neuroticism |  |  | -0.045 | (0.085) |  |  | -0.198^**^ | (0.051) |
| Wid. x extraversion |  |  | -0.023 | (0.090) |  |  | 0.146^*^ | (0.063) |
| Wid. x conscientiousness |  |  | 0.160 | (0.085) |  |  | 0.004 | (0.055) |
| Wid. x openness |  |  | -0.087 | (0.082) |  |  | -0.042 | (0.056) |
| Wid. x agreeableness |  |  | -0.018 | (0.077) |  |  | -0.253^**^ | (0.055) |
| Constant | 7.257^**^ | (0.005) | 7.257^**^ | (0.005) | 7.232^**^ | (0.004) | 7.232^**^ | (0.004) |
| Observations | 112877 |  | 112877 |  | 117861 |  | 117861 |  |
| Individuals | 11077 |  | 11077 |  | 11798 |  | 11798 |  |
| Log likelihood | -172301 |  | -172304 |  | -183226 |  | -183207 |  |

Standardized variables; standard errors in parentheses

^*^ *p* < 0.05, ^**^ *p* < 0.01

^a^ For years in which individuals cohabit

^b^ Main effects of personality traits not estimated since time-constant.

Source: SOEP 2018 (waves 1991-2018, unweighted); own calculations

Table A.12. Fixed effects regression of life satisfaction: the effect of cohabitation start^a^ by personality traits, excluding lesbian and gay couples

|  | Men  (1) |  | Women  (2) |  |
| --- | --- | --- | --- | --- |
| Age | -0.294^**^ | (0.033) | -0.026 | (0.028) |
| Cohabitation | 0.344^**^ | (0.039) | 0.370^**^ | (0.036) |
| Cohab. x neuroticism^b^ | -0.007 | (0.038) | 0.045 | (0.035) |
| Cohab. x extraversion | -0.071 | (0.038) | 0.055 | (0.036) |
| Cohab. x conscientiousness | 0.049 | (0.036) | 0.053 | (0.035) |
| Cohab. x openness | 0.018 | (0.041) | -0.041 | (0.036) |
| Cohab. x agreeableness | -0.045 | (0.038) | 0.023 | (0.036) |
| Constant | 6.801^**^ | (0.020) | 6.908^**^ | (0.008) |
| Observations | 25356 |  | 35591 |  |
| Individuals | 3439 |  | 4777 |  |
| Log likelihood | -39982.6 |  | -57499.0 |  |

Standardized variables; standard errors in parentheses

^*^ *p* < 0.05, ^**^ *p* < 0.01

^a^ For years in which individuals do not cohabit

^b^ Main effects of personality traits not estimated since time-constant.

Source: SOEP 2018 (waves 1991-2018, unweighted); own calculations

Table A.13. Fixed effects regression of life satisfaction: the effects of separation and widowhood^a^ by personality traits, excluding lesbian and gay couples

|  | Men  (1) |  | (2) |  | Women  (3) |  | (4) |  |
| --- | --- | --- | --- | --- | --- | --- | --- | --- |
| Age | -0.274^**^ | (0.011) | -0.274^**^ | (0.011) | -0.280^**^ | (0.012) | -0.280^**^ | (0.012) |
| Separation | -0.784^**^ | (0.039) | -0.760^**^ | (0.037) | -0.530^**^ | (0.036) | -0.522^**^ | (0.033) |
| Widowhood | -1.288^**^ | (0.071) | -1.343^**^ | (0.078) | -1.351^**^ | (0.047) | -1.182^**^ | (0.055) |
| Sep. x neurotiscm^b^ | -0.095^*^ | (0.038) |  |  | -0.021 | (0.034) |  |  |
| Sep. x extraversion | -0.002 | (0.041) |  |  | 0.002 | (0.036) |  |  |
| Sep. x conscientiousness | 0.080^*^ | (0.040) |  |  | 0.081^*^ | (0.036) |  |  |
| Sep. x openness | 0.010 | (0.040) |  |  | 0.036 | (0.035) |  |  |
| Sep. x agreeableness | -0.036 | (0.038) |  |  | -0.012 | (0.035) |  |  |
| Wid. x neuroticism |  |  | -0.059 | (0.084) |  |  | -0.214^**^ | (0.049) |
| Wid. x extraversion |  |  | 0.019 | (0.088) |  |  | 0.147^*^ | (0.060) |
| Wid. x conscientiousness |  |  | 0.085 | (0.080) |  |  | 0.016 | (0.053) |
| Wid. x openness |  |  | -0.134 | (0.080) |  |  | -0.029 | (0.054) |
| Wid. x agreeableness |  |  | 0.023 | (0.076) |  |  | -0.259^**^ | (0.053) |
| Constant | 7.265^**^ | (0.004) | 7.265^**^ | (0.004) | 7.252^**^ | (0.003) | 7.252^**^ | (0.003) |
| Observations | 125429 |  | 125429 |  | 132640 |  | 132640 |  |
| Individuals | 12628 |  | 12628 |  | 13574 |  | 13574 |  |
| Log likelihood | -192129 |  | -192133 |  | -206249 |  | -206224 |  |

Standardized variables; standard errors in parentheses

^*^ *p* < 0.05, ^**^ *p* < 0.01

^a^ For years in which individuals cohabit

^b^ Main effects of personality traits not estimated since time-constant.

Source: SOEP 2018 (waves 1991-2018, unweighted); own calculations

Figure A.1. Predicted life satisfaction by time before and after relationship transition (derived from fixed effects models controlling for age)
